# Case Report: From misdiagnosis to successful treatment using mepolizumab in ANCA-negative severe EGPA—clinical lessons in eosinophil-targeted therapy

**DOI:** 10.3389/fmed.2026.1881722

**Published:** 2026-07-08

**Authors:** Ju-Zhang Li, Si-Yao Guan, Qiao-Zhen Wu, Jiang-Nan Zheng

**Affiliations:** 1 Department of Respiratory and Critical Care Medicine, Suzhou Ninth Hospital Affiliated to Soochow University (Suzhou Ninth People's Hospital), Suzhou, China; 2 Department of Anesthesiology, Suzhou Ninth Hospital Affiliated to Soochow University (Suzhou Ninth People's Hospital), Suzhou, China

**Keywords:** ANCA-negative, asthma, eosinophilic granulomatosis with polyangiitis, mepolizumab, myocarditis

## Abstract

This paper presents the complex diagnostic course and treatment of a 33-year-old female patient with anti-neutrophil cytoplasmic antibodies (ANCA)-negative eosinophilic granulomatosis with polyangiitis (EGPA). The patient initially presented with asthma and chronic rhinosinusitis. After treatment with omalizumab and glucocorticoids, she progressed to severe EGPA with multi-organ involvement. Following discontinuation of omalizumab and initiation of mepolizumab (300 mg every 4 weeks), her symptoms resolved, and glucocorticoids were tapered. This case suggests that mepolizumab could be effective for inducing and maintaining remission in ANCA-negative severe EGPA with multi-organ involvement. Exposure to omalizumab in undiagnosed EGPA may either mask or unmask disease progression. Clinicians should carefully evaluate for EGPA before initiating biologic therapy in patients with asthma. Furthermore, we successfully extended the mepolizumab dosing interval to 8 weeks with sustained remission, providing preliminary, hypothesis-generating evidence that interval extension of MEP may be considered as a potential de-escalation strategy for maintenance therapy in stable EGPA.

## Background

Eosinophilic granulomatosis with polyangiitis (EGPA) is a rare systemic necrotizing vasculitis associated with eosinophilia that often affects the respiratory tract, heart, nervous system, and skin. Anti-neutrophil cytoplasmic antibodies (ANCA)-negative patients more often develop severe cardiac and pulmonary involvement and have a relatively worse prognosis. In its early stage, ANCA-negative EGPA is often misdiagnosed as refractory asthma and may be treated with biologic agents, such as anti-immunoglobulin E monoclonal antibodies. However, this treatment carries the risk of masking the progression of systemic vasculitis. Mepolizumab (MEP) is a humanized anti-interleukin-5 monoclonal antibody that specifically reduces eosinophil levels and has been recommended for the treatment of EGPA, as is benralizumab (1). However, before December 2025, mepolizumab was the only anti-interleukin-5 agent approved for EGPA in China. Its application experience in ANCA-negative, severe EGPA with multi-organ involvement remains limited.

## Case report

The patient, a 33-year-old female, had a history of allergic rhinitis for 8 years and a history of recurrent wheezing for 1 year. She first visited the hospital in November 2022 due to an asthma attack. Physical examination showed bilateral wheezing. Blood tests showed the proportion of eosinophils (EOS) was 9.8%, the eosinophil count was 680 cells/μL, and the total serum immunoglobulin E (IgE) was 330 IU/L. Pulmonary function tests revealed a Forced Expiratory Volume in 1 s/Forced Vital Capacity (FEV1/FVC) ratio of 82%, FEV1 of 85% of the predicted value, and a 12% improvement in FEV1 (220 mL) following bronchodilator inhalation. Fractional exhaled nitric oxide (FeNO) was 152 ppb. Chest computed tomography (CT) was normal, but sinus CT showed pansinusitis. She was diagnosed with acute exacerbation of asthma, allergic rhinitis, and chronic rhinosinusitis. Her symptoms improved after treatment with the budesonide (320 μg)/formoterol fumarate (9 μg) combination inhaler.

In April 2023, she was hospitalized for a severe asthma exacerbation. Blood tests showed an EOS percentage of 23% and an absolute EOS count of 1920 cells/μL. The total IgE increased to 1710 IU/L. The IgE and immunoglobulin G (IgG) antibodies of *Aspergillus fumigatus* were negative. ANCA was negative. After intravenous methylprednisolone and nebulization therapy, she improved and was discharged. At discharge, the patient was replaced with the salmeterol (50 μg)/fluticasone propionate (500 μg) combination inhaler, supplemented with budesonide nasal spray. Besides, the patient’s allergic rhinitis began in childhood and was diagnosed 8 years prior, followed by asthma, a typical allergic progression. As mepolizumab had not yet been included in the local health insurance reimbursement framework at that time, omalizumab (600 mg every 2 weeks) was chosen for asthma control. The patient’s asthma symptoms were well controlled, with asthma control test (ACT) scores maintained at 22–25 and no acute exacerbations observed during outpatient follow-up visits.

**Figure 1:**
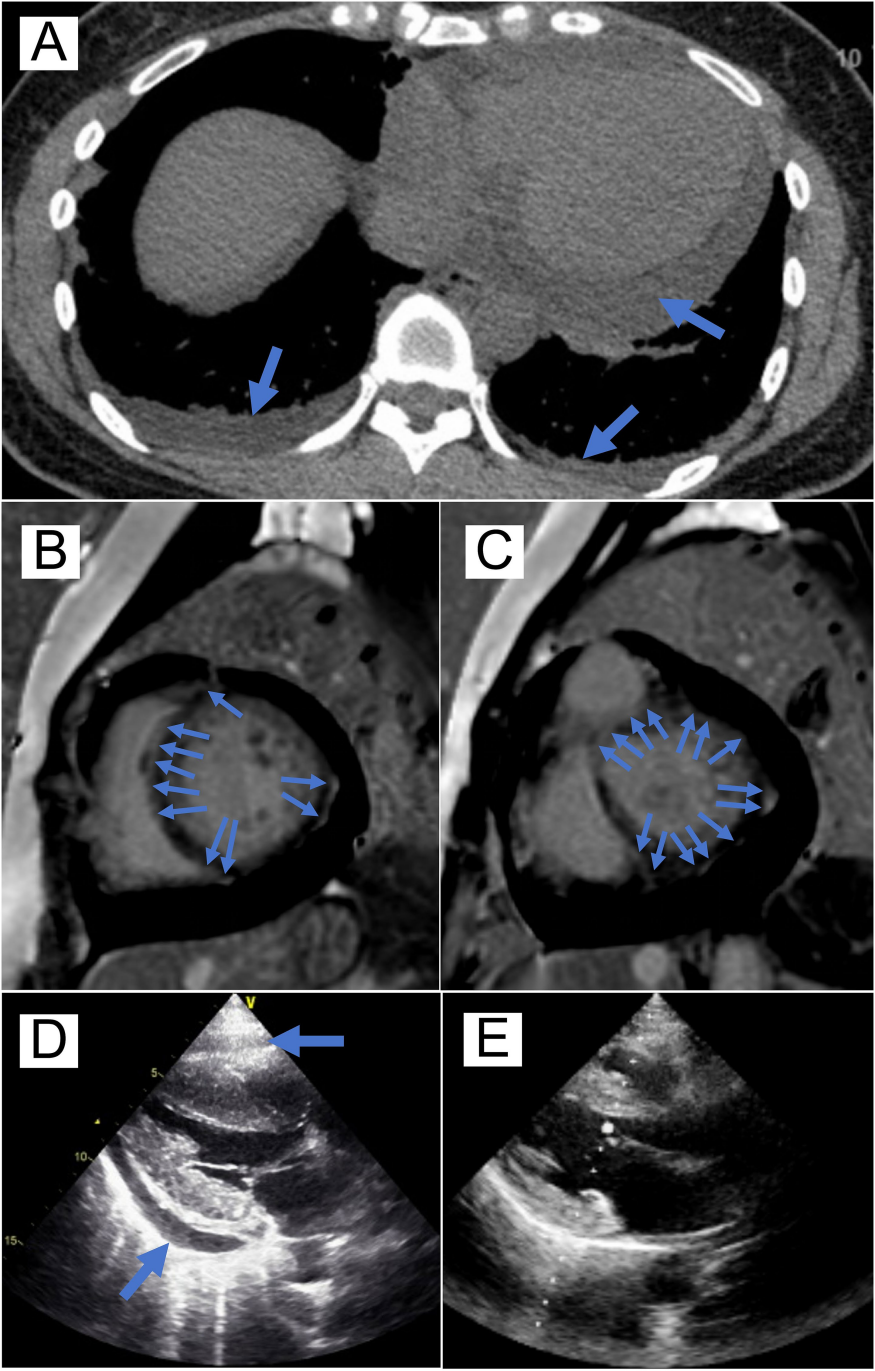
Arrows indicated bilateral pleural and pericardial effusions on chest CT **(A)**. Cardiac MRI with late gadolinium enhancement: arrows indicate patchy non-ischemic subepicardial enhancement in the mid-ventricular segment **(B)** and basal segment **(C)** of the left ventricle, consistent with myocarditis. Echocardiography showed left and right ventricular wall thickening. Arrows indicated moderate pericardial effusion **(D)**. A follow-up echocardiogram was performed in October 2024: the LVEF increased to 54%, and no pericardial effusion was found **(E)**.

In January 2024, the patient was hospitalized for chest pain. The electrocardiogram showed ST segment depression in some leads, and troponin I was 7.53 ng/mL (reference range 0–0.1 ng/mL). Coronary artery CT angiography indicated no abnormalities in the coronary arteries. Chest CT showed bilateral pleural and pericardial effusions (Figure 1A). Cardiac late gadolinium enhancement (LGE) MRI imaging demonstrated patchy non-ischemic subepicardial enhancement in the mid-ventricular and basal segments of the left ventricle, findings consistent with myocardial inflammation and supporting the diagnosis of myocarditis (Figures 1B,C). Cardiac ultrasound showed left and right ventricular wall thickening, moderate pericardial effusion (Figure 1D), and left ventricular ejection fraction (LVEF) 65%. Laboratory tests revealed an EOS percentage of 49.3% and an absolute EOS count of 9,230 cells/μL. The patient denied a definite history of consuming raw or undercooked meat or seafood, as well as any close contact history; three consecutive stool examinations revealed no ova or parasites. Screening for autoantibodies and viral infections was negative, and serum IgG4 levels were within the normal range. Bone marrow biopsy showed no morphological abnormalities, and DNA sequencing and immunophenotyping for hematologic malignancy were negative. The patient was diagnosed with eosinophilia, myocarditis, and pericardial effusion. Following oral administration of methylprednisolone (initial dose 32 mg/day), the patient’s chest pain resolved, and troponin I levels normalized. Methylprednisolone was gradually reduced to 12 mg/day for maintenance. Follow-up echocardiography in February 2024 showed a very small amount of pericardial effusion and a LVEF of 70%.

In May 2024, the patient developed chest tightness and fatigue, accompanied by numbness of both lower limbs and intermittent abdominal pain. Troponin I and creatine kinase-MB (CK-MB) were within normal limits. Echocardiography showed decreased left ventricular function (LVEF 41%) and a small amount of pericardial effusion (8 mm on the left ventricular lateral wall). Electromyography (EMG) showed abnormal conduction of the left common peroneal nerve and tibial nerve, consistent with mononeuritis multiplex. Abdominal CT was normal. Despite lacking histopathological evidence, according to the EGPA classification criteria established by the 2022 ACR/EULAR (2), she was diagnosed with EGPA (systemic, severe) after multidisciplinary discussion, with a Birmingham Vasculitis Activity Score (BVAS) of 18. Summary of the 2022 ACR/EULAR classification criteria and individual items applied to the present case are shown in Table 1. The decision not to perform a biopsy was based on the following considerations. First, current guidelines indicate that while biopsy is recommended when feasible, it is not essential for the diagnosis of EGPA when typical clinical features are present. Second, although the patient had pansinusitis, biopsies of the sinonasal mucosa are often non-diagnostic for EGPA due to non-specific inflammatory changes. Third, endomyocardial biopsy was not available at our institution and carries procedural risks that outweigh its diagnostic benefit in this clinical context. Thus, there were no suitable lesions for biopsy with an acceptable risk-to-diagnostic-yield ratio.

**Table 1:** 2022 ACR/EULAR EGPA classification criteria* and patient scoring.

Criteria	Weight	Patient’s finding	Score
Obstructive airway disease	+3	Asthma	3
Nasal polyps	+3	Pansinusitis on CT	3
Mononeuritis multiplex	+1	EMG: abnormal conduction of left common peroneal/tibial nerve	1
Blood eosinophil count ≥1,000/μL	+5	Blood eosinophil count 9,230/μL	5
Extravascular eosinophilic-predominant inflammation on biopsy	+2	Absent	0
Positive test for cANCA or anti-PR3 antibodies	−3	Negative	0
Hematuria	−1	Negative	0
Total			12

EMG, Electromyography; cANCA, cytoplasmic antineutrophil cytoplasmic antibodies; anti-PR3, antiproteinase 3; * A total score ≥ 6 points classifies a patient as having EGPA with 85% sensitivity and 99% specificity.

Although corticosteroids combined with cyclophosphamide is the standard first-line regimen for cardiac involvement in EGPA, several factors influenced our decision. Prior to diagnosis, the patient had received glucocorticoids for approximately 6 months (32 mg/day tapered to 12 mg/day of methylprednisolone), resulting in significant cumulative steroid exposure. She strongly opposed further dose escalation due to side effects. The cardiac deterioration at diagnosis (LVEF 70 to 41%) occurred without elevated troponin I or CK-MB, suggesting chronic myocardial damage rather than acute inflammation. Given her partial prior response to steroids, childbearing age, and these contextual factors, we added mepolizumab as salvage therapy without further increasing the steroid dose, under close monitoring. From June 2024, omalizumab was discontinued, and MEP 300 mg subcutaneous injection was added once every 4 weeks. After treatment, the patient’s symptoms improved rapidly, chest tightness, fatigue and limb numbness were alleviated, and asthma symptoms were well controlled. Methylprednisolone was gradually reduced. A follow-up echocardiogram was performed in October 2024: the LVEF increased to 54%, and no pericardial effusion was found (Figure 1E).

**Figure 2:**
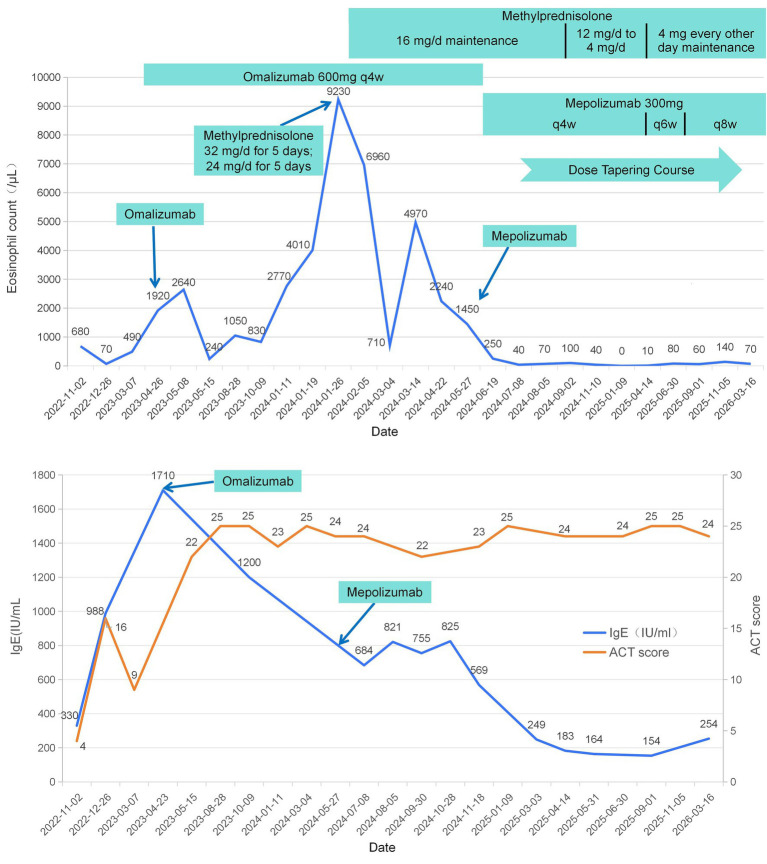
Summary of treatment and changing trends of EOS count, serum total IgE, and ACT scores over time. Omalizumab: from April 2023 to June 2024. Mepolizumab: started June 2024; extended to every 6 weeks in January 2025, and to every 8 weeks in April 2025. Methylprednisolone: 32 mg/d for 5 days, then 24 mg/d for 5 days (January 2024); 16 mg/d maintenance (February 2024); tapered to 12 mg/d (August 2024), 8 mg/d (October 2024), 6 mg/d (December 2024), 4 mg/d (February 2025), and 4 mg every other day (April 2025). ACT scores are shown as annotations at corresponding time points, ranging from 22 to 25 during omalizumab and mepolizumab treatment, indicating good asthma control.

**Figure 3:**
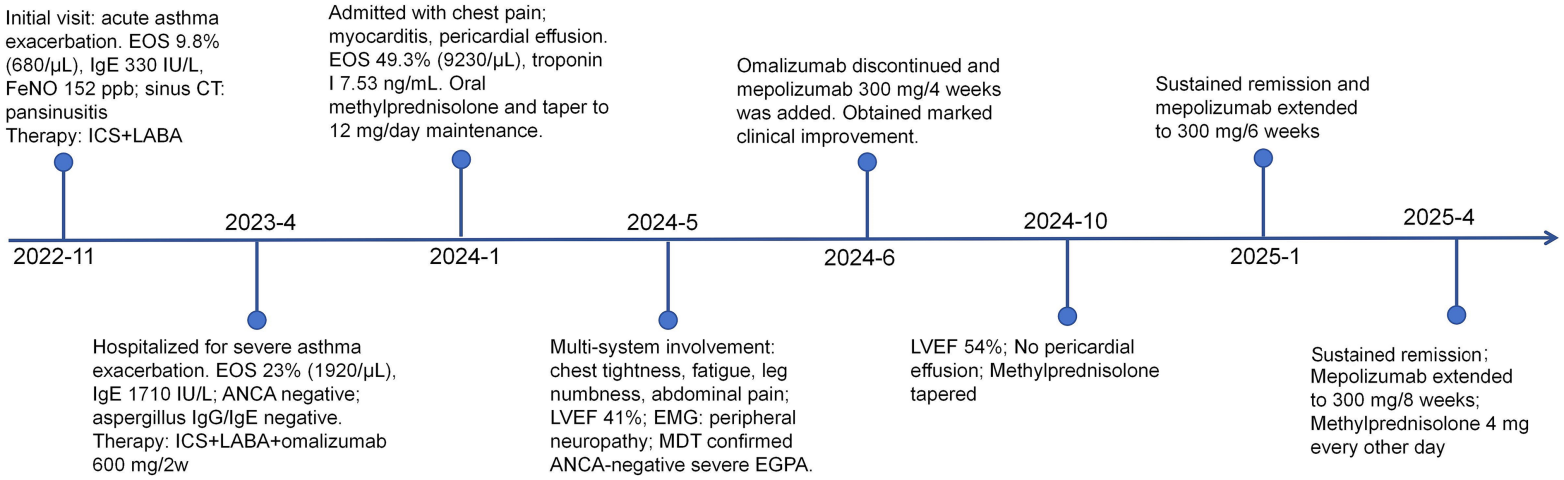
The timeline of the patient’s diagnosis and treatment course. ICS, Inhaled Corticosteroids; LABA, Long-acting β₂-adrenergic agonist.

Because the patient achieved remission (defined as BVAS of 0 on a methylprednisolone dose ≤ 6 mg/day, equivalent to 7.5 mg/day of prednisone) and expressed preference for reduced treatment frequency to lower healthcare burden, we attempted stepwise extension of mepolizumab intervals. First extension (4 weeks to 6 weeks): In January 2025, after the patient had maintained remission for 7 months on mepolizumab 300 mg every 4 weeks, meeting guideline-defined remission criteria, with BVAS = 0 and oral methylprednisolone maintained at 6 mg/day. During follow-up, asthma was well-controlled (ACT score 22–25) and the peripheral blood eosinophil count was persistently within 300/μL. So, we extended the dosing interval to 300 mg every 6 weeks. Second extension (6 weeks to 8 weeks): The patient remained stable on the 6-week regimen, with BVAS = 0, oral methylprednisolone maintained at 4 mg/day, ACT score 23–25, and eosinophil count <100/μL. Therefore, in April 2025, we further extended the interval to 300 mg every 8 weeks, and methylprednisolone was tapered to 4 mg every other day. Follow-up: During follow-up through March 2026 (12 months after the 8-week interval was initiated), the patient has maintained sustained remission with BVAS = 0, oral methylprednisolone maintained at 4 mg every other day, ACT score 24–25, and eosinophil count persistently <100/μL. Table 2 shows the treatment timeline, glucocorticoid tapering, and clinical parameters during mepolizumab dose interval extension. Figure 2 shows the treatment and changing trends of EOS count, serum total IgE, and ACT scores over time. Figure 3 shows the timeline of the patient’s diagnosis and treatment course.

**Table 2:** Treatment timeline, glucocorticoid tapering, and clinical parameters during mepolizumab dose interval extension.

Parameter	MEP interval	Duration (month)	BVAS	MP dose	ACT score	EOS count (μL)	Remission
Eosinophilia, myocarditis (Jan 2024)	NA	NA	NA	32 mg/day for 5 days; 24 mg/day for 5 days; 16 mg/day maintenance	23	9,230	Negative
First Administration (June 2024)	300 mg/4 weeks	NA	18	16 mg/day maintenance	24	1,450	Negative
Maintenance (Aug 2024)	300 mg/4 weeks	2	7	12 mg/day maintenance	22–23	<300	Negative
Maintenance (Oct 2024)	300 mg/4 weeks	4	0	8 mg/day maintenance	22–23	<100	Negative
Maintenance (Dec 2024)	300 mg/4 weeks	6	0	6 mg/day maintenance	23	<100	Remission
First Extension (Jan 2025)	300 mg/4 weeks to 300 mg/6 weeks	7*	0	6 mg/day	22–25	<100	Remission
Maintenance (Feb 2025)	300 mg/6 weeks	2	0	4 mg/day	25	<100	Remission
Second extension (Apr 2025)	300 mg/6 weeks to 300 mg/8 weeks	3*	0	4 mg every other day	23–25	<100	Remission
Follow-up (Mar 2026)	300 mg/8 weeks (maintained)	12*	0	4 mg every other day	24–25	<200	Remission

NA, Not Applicable; MEP, Mepolizumab; MP, Methylprednisolone; BVAS, Birmingham Vasculitis Activity Score; ACT, Asthma Control Test; EOS, Eosinophil. * The patient remained on 300 mg/4 weeks for 7 months, 300 mg/6 weeks for 3 months, and 300 mg/8 weeks for 12 months of follow-up.

## Discussion

This case shows a severe EGPA patient with ANCA negative and multi-organ involvement who achieved significant relief after adding MEP. The evolution of this patient’s disease is typical: from prodromal respiratory symptoms such as asthma and sinusitis to life-threatening cardiac (myocarditis, pericardial effusion) and nervous system (peripheral neuropathy) involvement, with eosinophil levels paralleling disease activity. Without histopathological confirmation, EGPA was diagnosed based on the 2022 ACR/EULAR criteria (2). Given the marked eosinophilia and multi-organ involvement, broad differentials were considered. Allergic bronchopulmonary aspergillosis (ABPA) was excluded by negative Aspergillus serology and absence of central bronchiectasis. Hypereosinophilic syndrome (HES) was unlikely due to no thromboembolic events, no clonal eosinophilia on bone marrow biopsy, and DNA sequencing (including PDGFRA/B, and FGFR1 rearrangements). Hematologic malignancy was ruled out by normal bone marrow morphology, flow cytometry, and DNA sequencing. The patient had no raw seafood/meat exposure history, and three stool examinations were negative for ova/parasites, making parasitic infection unlikely. IgG4-related disease was excluded by normal serum IgG4 levels and lack of characteristic imaging. Thus, EGPA remained the most consistent diagnosis.

Although previous reports of successful use of omalizumab in EGPA (3–5), its safety in EGPA patients is still controversial (3, 6, 7). In our case, omalizumab effectively controlled asthma symptoms, as evidenced by ACT scores of 22–25 and the absence of acute exacerbations during follow-up. However, we subsequently observed an increase in the blood eosinophil count and systemic vasculitis activity. This observation aligns with previous reports suggesting that anti-IgE or anti-IL-5/IL-5R biologics may alter the classic presentation of EGPA. Previous reports by Fargeas et al. and Caminati et al. suggested that omalizumab may either mask or unmask the occurrence or progression of EGPA (6, 7). Ohmura et al. described potential masking of new-onset or relapsed EGPA during benralizumab treatment (8). Similarly, Saito et al. recently reported a case of EGPA emerging during benralizumab therapy without marked eosinophilia, which was successfully managed by switching to mepolizumab (9). Yukishima et al. also successfully switched a patient from benralizumab to mepolizumab in refractory EGPA with multi-organ dysfunction (10). Collectively, these reports, together with our case, indicate that biologics targeting type 2 inflammation may complicate EGPA diagnosis by suppressing typical features such as asthma and eosinophilia. This suppression can have dual effects: it may mask disease progression by improving asthma symptoms, or conversely, unmask EGPA by permitting glucocorticoid tapering. Furthermore, switching between biologics (e.g., from omalizumab or benralizumab to mepolizumab) may still achieve disease control, a strategy that warrants further investigation.

While high-dose glucocorticoid pulses combined with immunosuppressants (such as cyclophosphamide) was the first choice for severe EGPA (1), this approach carries significant risks, including immunosuppression and teratogenicity. Given the reproductive toxicity of cyclophosphamide and the fact that it does not always achieve complete symptom control, its use may be less favorable in younger patients, and current guidelines acknowledge that treatment decisions should be appropriately modified in special populations, particularly children and women of childbearing age (11). Kasuya et al. (12) reported a case in which a perinatal patient with EGPA who failed to respond to cyclophosphamide pulse therapy and tacrolimus was successfully switched to mepolizumab for disease control. Similarly, Morejón et al. (13) described mepolizumab as an effective alternative to immunosuppressive and teratogenic therapies for the treatment of severe EGPA with cardiac involvement. These considerations are especially relevant in younger patients to preserve fertility. This case involves women of childbearing age. MEP can save glucocorticoids while avoiding immunosuppression and teratogenic risks, which is particularly significant in this patient population. Besides, the patient had already initiated systemic glucocorticoid therapy approximately 6 months before the confirmed diagnosis of EGPA due to her marked eosinophilia. By the time EGPA was diagnosed, several multi-organ involvement symptoms, including myocarditis, had partially resolved with glucocorticoid treatment. Therefore, we selected mepolizumab as a further induction therapy to enable continued glucocorticoid tapering while maintaining disease control.

Although MEP 300 mg every 4 weeks is the standard regimen for inducing remission in EGPA (14, 15), the dosage and timing of MEP maintenance treatment during the remission period of EGPA are still inconclusive. Studies have shown that MEP 100 mg/4 weeks can effectively control the asthma symptoms of EGPA and prevent the recurrence of vasculitis (16, 17). A study by Moroni et al. believes that downgrading MEP from 300 mg/4 weeks to 100 mg/4 weeks is still effective in maintaining systemic remission of EGPA, but 50% of patients still have EGPA-related sinus symptom recurrence and require increased dose or local treatment (18). Another report shows that the use of low-dose MEP in high-risk EGPA patients is insufficient to prevent the recurrence of permanent organ damage (19). These findings suggest that dose reduction may not be optimal for all patients, particularly those with prior severe cardiac involvement like our case.

Compared with reducing the dose, extending the dosing interval is also a common way of downgrading biological therapy. For patients with well-controlled SEA, extending mepolizumab dosing intervals to 6–8 weeks maintains asthma stability and may reduce healthcare costs (20, 21). However, until recently, studies on the efficacy of extended dosing intervals of MEP for maintaining remission in EGPA were lacking. A retrospective cohort study reported in 2026 by Ayano et al. evaluated mepolizumab injection spacing in 43 EGPA patients, including 10 who underwent interval extension to 5 weeks (*n* = 7) or 6 weeks (*n* = 3) (22). That study found no statistically significant difference in time to relapse or drug discontinuation between standard and spacing intervals, suggesting that spacing is a viable and well-tolerated strategy for stable EGPA. Building on the findings of Ayano et al. (22), which demonstrated the feasibility of spacing to 5–6 weeks, we successfully extended MEP dosing intervals to 8 weeks in a high-risk, ANCA-negative severe EGPA patient with severe cardiac involvement, a longer extension than previously reported. The remission was maintained up to 12 months of follow-up, providing preliminary, hypothesis-generating evidence that exploring individualized and more cost-effective maintenance programs for EGPA may be warranted. This case extends the existing literature by suggesting that even longer intervals (8 weeks) may be feasible in carefully selected, stable patients. However, given the single-case nature of this report, the feasibility and safety of this approach require further validation. Importantly, the risk of vasculitis relapse or progression cannot be excluded and warrants careful monitoring. Any attempt at interval extension should therefore be accompanied by close surveillance of clinical symptoms, peripheral blood EOS counts, and organ function.

A limitation of this case is the lack of histopathological evidence for eosinophilic vasculitis and the absence of cytokine profiling, eosinophil phenotyping, or tissue immunology. However, its typical clinical evolution and significant response to specific targeted therapy strongly support the diagnosis of EGPA. As a single uncontrolled case report, the findings presented here are hypothesis-generating rather than generalizable. Larger prospective studies are needed to validate the safety and efficacy of mepolizumab interval extension in EGPA.

We acknowledge that cyclophosphamide with high-dose glucocorticoids remains the standard of care for severe EGPA with cardiac involvement. In this case, mepolizumab was selected due to unique circumstances—namely, prolonged pre-diagnostic steroid exposure, patient preference against further dose escalation, and the absence of acute myocardial injury markers at the time of cardiac deterioration. This approach should not detract from established guidelines; rather, it underscores the importance of early EGPA screening in patients with eosinophilic asthma, especially before initiating type 2-targeting biologics, which require careful evaluation. Furthermore, it suggests that mepolizumab may be considered as an alternative in carefully selected scenarios where standard therapy is less feasible.

## Conclusion

Clinicians should maintain vigilance in screening for EGPA among patients with eosinophilic asthma and rigorously evaluate the suitability of omalizumab. MEP reduces glucocorticoid requirements and avoids the immunosuppression and teratogenicity of cyclophosphamide, an advantage for women of childbearing age, although human data on mepolizumab safety during pregnancy remain limited. Notably, in this case, sustained remission (BVAS of 0 on a methylprednisolone dose of 4 mg every other day) was maintained up to 12 months after gradually extending the dosing interval from 4 weeks to 8 weeks. This observation provides preliminary, hypothesis-generating evidence that interval extension may be explored as a potential de-escalation strategy for maintenance therapy in stable EGPA. If confirmed in future studies, such an approach could help reduce medical costs while maintaining disease control. However, the effectiveness and safety of this strategy require further investigation.

## Data Availability

The original contributions presented in the study are included in the article/supplementary material, further inquiries can be directed to the corresponding authors.

## References

[1] Hellmich B Sanchez-Alamo B Schirmer JH Berti A Blockmans D Cid MC . EULAR recommendations for the management of ANCA-associated vasculitis: 2022 update. Ann Rheum Dis. (2024) 83:30–47. doi: 10.1136/ard-2022-223764, 36927642

[2] Grayson PC Ponte C Suppiah R Robson JC Craven A Judge A . American College of Rheumatology/European Alliance of associations for rheumatology classification criteria for eosinophilic granulomatosis with polyangiitis. Arthritis Rheumatol. (2022) 74:386–92. doi: 10.1136/ard-2022-223482 35106968

[3] Basta F Mazzuca C Nucera E Schiavino D Afeltra A Antonelli Incalzi R. Omalizumab in eosinophilic granulomatosis with polyangiitis: friend or foe? A systematic literature review. Clin Exp Rheumatol. (2020) 38:214–20. 32083537

[4] Celebi Sozener Z Gorgulu B Mungan D Sin BA Misirligil Z Aydin O . Omalizumab in the treatment of eosinophilic granulomatosis with polyangiitis (EGPA): single-center experience in 18 cases. World Allergy Organ J. (2018) 11:39. doi: 10.1186/s40413-018-0217-0, 30524647 PMC6276141

[5] Detoraki A Di Capua L Varricchi G Genovese A Marone G Spadaro G. Omalizumab in patients with eosinophilic granulomatosis with polyangiitis: a 36-month follow-up study. J Asthma. (2016) 53:201–6. doi: 10.3109/02770903.2015.1081700, 26377630

[6] Fargeas M Devouassoux G Gerfaud-Valentin M. Onset of eosinophilic granulomatosis with polyangiitis (EGPA) after anti-Th2 biotherapy initiation in severe asthma patients: report of 3 cases. Respir Med Res. (2024) 85:101070. doi: 10.1016/j.resmer.2023.101070 38141578

[7] Caminati M Fassio A Alberici F Baldini C Bello F Cameli P . Eosinophilic granulomatosis with polyangiitis onset in severe asthma patients on monoclonal antibodies targeting type 2 inflammation: report from the European EGPA study group. Allergy. (2024) 79:516–9. doi: 10.1111/all.15934, 37904674

[8] Ohmura SI Yonezawa H Ohkubo Y. Potential masking of new-onset or relapsed eosinophilic granulomatosis with polyangiitis during benralizumab treatment: a case series. J Allergy Clin Immunol Glob. (2025) 4:100551. doi: 10.1016/j.jacig.2025.100551, 40895411 PMC12396454

[9] Yukishima T Yonezawa H Aono Y Yamaguchi K Otsuki Y Ohmura SI. Successful switching treatment of mepolizumab for refractory eosinophilic granulomatosis with polyangiitis and multiple organ dysfunction under benralizumab treatment: a case report. Mod Rheumatol Case Rep. (2025) 9:8. doi: 10.1093/mrcr/rxaf008, 39856496

[10] Saito Y Ito J Watanabe T Tanabe Y Sasano H Sato Y . A case of eosinophilic granulomatosis with Polyangiitis emerging during Benralizumab therapy: successful management through a switch to Mepolizumab therapy. J Asthma Allergy. (2026) 19:568930. doi: 10.2147/JAA.S568930, 41778059 PMC12951869

[11] Emmi G Bettiol A Gelain E Bajema IM Berti A Burns S . Evidence-based guideline for the diagnosis and management of eosinophilic granulomatosis with polyangiitis. Nat Rev Rheumatol. (2023) 19:378–93. doi: 10.1038/s41584-023-00958-w, 37161084

[12] Kasuya A Kitano S Hoshino T Ishibe JI Imura K Goto H . Successful control of severe eosinophilic granulomatosis with polyangiitis in a pregnancy and perinatal period: a use of mepolizumab. J Dermatol. (2019) 46:e309–11. doi: 10.1111/1346-8138.14869, 30932239

[13] Morejon L Quirce S Dominguez-Ortega J Romero D Noblejas A Rios-Blanco JJ . Mepolizumab as an effective alternative to immunosuppressive and teratogenic therapies for the early treatment of EGPA: a case report. J Investig Allergol Clin Immunol. (2025) 35:389–91. doi: 10.18176/jiaci.1087, 40440108

[14] Wechsler ME Akuthota P Jayne D Khoury P Klion A Langford CA . Mepolizumab or placebo for eosinophilic granulomatosis with Polyangiitis. N Engl J Med. (2017) 376:1921–32. doi: 10.1056/NEJMoa1702079, 28514601 PMC5548295

[15] Spataro F Cacciapaglia F Carlucci P Solimando AG Desantis V Di Girolamo A . Efficacy and safety of mepolizumab 300 mg in eosinophilic granulomatosis with polyangiitis: a meta-analysis of eight retrospective studies. Allergy. (2025) 80:2660–2. doi: 10.1111/all.16625, 40560689

[16] Akdime F Puechal X Roche N. Mepolizumab in patients with eosinophilic granulomatosis with polyangiitis in remission: what is the right dose? J Allergy Clin Immunol Pract. (2021) 9:2942–3. doi: 10.1016/j.jaip.2021.03.061, 34246444

[17] Caminati M Crisafulli E Lunardi C Micheletto C Festi G Maule M . Mepolizumab 100 mg in severe asthmatic patients with EGPA in remission phase. J Allergy Clin Immunol Pract. (2021) 9:1386–8. doi: 10.1016/j.jaip.2020.09.025, 33011303

[18] Moroni L Batani V Gallina GD Benanti G Cilona M Cariddi A . Step-down treatment with mepolizumab for eosinophilic granulomatosis with polyangiitis: a real-life single-Centre study. Rheumatology (Oxford). (2025) 64:5108–11. doi: 10.1093/rheumatology/keaf201, 40221861

[19] Vanthuyne A Riemann S Brusselle G. Relapse of eosinophilic granulomatosis with polyangiitis (EGPA) despite maintenance treatment with low-dose mepolizumab. Respirol Case Rep. (2025) 13:e70186. doi: 10.1002/rcr2.70186, 40337294 PMC12056496

[20] Bölke G Tong X Zuberbier T Bousquet J Bergmann KC. Extension of mepolizumab injection intervals as potential of saving costs in well controlled patients with severe eosinophilic asthma. World Allergy Organ J. (2022) 15:100703. doi: 10.1016/j.waojou.2022.100703, 36254185 PMC9527939

[21] Soendergaard MB Bjerrum AS Rasmussen LM Lock-Johansson S Hilberg O Hansen S . Titration of anti-IL-5 biologics in severe asthma: an open-label randomised controlled trial (the OPTIMAL study). Eur Respir J. (2024) 64:2400404. doi: 10.1183/13993003.00404-2024, 38843910 PMC11339407

[22] Ayano M Nishimura K Inoue Y Yoshimura M Nishimura N Kimoto Y . Effectiveness, tolerability, and safety of Mepolizumab injection spacing in patients with eosinophilic granulomatosis with Polyangiitis: a retrospective cohort study. J Clin Rheumatol. (2026) 32:85–90. doi: 10.1097/RHU.0000000000002321, 41614630

